# Values and options in cancer care (VOICE): study design and rationale for a patient-centered communication and decision-making intervention for physicians, patients with advanced cancer, and their caregivers

**DOI:** 10.1186/1471-2407-13-188

**Published:** 2013-04-09

**Authors:** Michael Hoerger, Ronald M Epstein, Paul C Winters, Kevin Fiscella, Paul R Duberstein, Robert Gramling, Phyllis N Butow, Supriya G Mohile, Paul R Kaesberg, Wan Tang, Sandy Plumb, Adam Walczak, Anthony L Back, Daniel Tancredi, Alison Venuti, Camille Cipri, Gisela Escalera, Carol Ferro, Don Gaudion, Beth Hoh, Blair Leatherwood, Linda Lewis, Mark Robinson, Peter Sullivan, Richard L Kravitz

**Affiliations:** 1 Rochester Healthcare Decision-Making Group, University of Rochester Medical Center, Rochester, New York, USA; 2 Department of Psychiatry, University of Rochester Medical Center, Rochester, New York, USA; 3 James P. Wilmot Cancer Center, University of Rochester Medical Center, Rochester, New York, USA; 4 Center for Communication and Disparities Research, Department of Family Medicine, University of Rochester Medical Center, Rochester, New York, USA; 5 Centre for Medical Psychology and Evidence-based Medicine, School of Psychology, The University of Sydney, Sydney, NSW, Australia; 6 Department of Internal Medicine, University of California, Davis, Sacramento, California, USA; 7 Department of Biostatistics and Computational Biology, University of Rochester Medical Center, Rochester, New York, USA; 8 Department of Medicine, University of Washington, Fred Hutchinson Cancer Research Center, Seattle, Washington, USA; 9 Center for Healthcare Policy and Research, University of California, Davis, Sacramento, California, USA; 10 University of Rochester Medical Center, 300 Crittenden Blvd, Rochester, NY, USA

**Keywords:** Patient-centered care, Decision making, End-of-life care, Communication, Caregivers, Cancer, Palliative care, Quality of life, Utilization, Physician-patient relations

## Abstract

**Background:**

Communication about prognosis and treatment choices is essential for informed decision making in advanced cancer. This article describes an investigation designed to facilitate communication and decision making among oncologists, patients with advanced cancer, and their caregivers.

**Methods/design:**

The Values and Options in Cancer Care (VOICE) Study is a National Cancer Institute sponsored randomized controlled trial conducted in the Rochester/Buffalo, NY and Sacramento, CA regions. A total of 40 oncologists, approximately 400 patients with advanced cancer, and their family/friend caregivers (one per patient, when available) are expected to enroll in the study. Drawing upon ecological theory, the intervention uses a two-pronged approach: oncologists complete a multifaceted tailored educational intervention involving standardized patient instructors (SPIs), and patients and caregivers complete a coaching intervention to facilitate prioritizing and discussing questions and concerns. Follow-up data will be collected approximately quarterly for up to three years.

**Discussion:**

The intervention is hypothesized to enhance patient-centered communication, quality of care, and patient outcomes. Analyses will examine the effects of the intervention on key elements of physician-patient-caregiver communication (primary outcomes), the physician-patient relationship, shared understanding of prognosis, patient well-being, and health service utilization (secondary outcomes).

**Trial registration:**

Clinical Trials Identifier: NCT01485627

## Background

Crafting care that is concordant with the patient’s wishes in the context of serious illness requires clear, patient-centered communication [1]. Most patients with advanced cancer (>80%) want frank yet sensitive discussions with their physicians about prognosis and treatment choices, and want to be involved and informed about decisions regarding their care, regardless of whether they wish to assume responsibility for making major health care decisions [2]. Yet, few actually have these discussions [3]. Consequently, patients often overestimate prognoses, underestimate disease severity, and have unrealistic expectations for cure [2-5]. Having frank, sensitive discussions is associated with more realistic prognostic estimates and decisions that are better aligned with patients’ wishes [3,5-8]. When these discussions occur before patients are critically ill, patients report greater well-being and have fewer unwanted aggressive interventions in the last weeks of life, with no detrimental effect on survival [3,5,9].

The VOICE (**V**alues and **O**ptions **I**n **C**ancer Car**e**) Study is a randomized controlled trial of a patient-centered communication intervention for oncologists, patients with advanced cancer, and their caregivers. Initial study findings are expected to be published in 2013, with follow-up complete in 2015. This article describes the empirical and theoretical rationale for the study, the tailored education and coaching communication intervention, the study measures and administration procedures, the planned analytic approach, and potential implications of this research.

### Patient-centered communication

VOICE targets important gaps in cancer communication research. Since the SUPPORT study [10], in which an ICU-based nurse intervention failed to influence care for critically ill patients with dire prognoses, there has been insufficient progress in improving clinical communication in the context of serious illness. The 2007 NCI monograph, *Patient*-*centered Communication and Cancer Care*[1], the Institute of Medicine [11], the American Society for Clinical Oncology, and the National Priorities Partnership all call for improvements in communication with patients who have serious and life-limiting illnesses, citing the effects of good communication on quality of care and quality of life.

Poor communication is common in advanced cancer, leading to healthcare decisions that are inadequately informed by patients’ preferences. Physicians often misjudge patients’ treatment preferences, desire for information, needs, and level of understanding [2,12]. Despite evidence that individuals with advanced cancer and their caregivers benefit from being informed about prognosis and treatment choices [1,2,13], physicians often intentionally overestimate survival [14] and avoid discussing prognosis until the patient has symptoms or there are no other treatments to offer, leading to inflated patient expectations about survival and the benefits of cytotoxic treatment [4,15]. Patients who have not discussed prognosis and treatment choices with their physicians are 3 to 8 times more likely to receive aggressive treatments in the last week of life [3,5], reducing physical and emotional quality of life and perhaps longevity [9]. Additionally, patients often alter their treatment choices when adequately informed [16]. Furthermore, although physicians and patients find prognostic discussions stressful, concealing the truth can be more harmful [17]. Indeed, evidence suggests that these discussions neither appear to cause harm nor diminish hope [18,19].

Communication about prognosis and treatment choices is essential for informed decision making in advanced cancer. Our intervention is designed to promote patient-centeredness [20], which is defined by the Institute of Medicine [21] as “care that is respectful of and responsive to individual patient preferences, needs, and values.” The intervention targets four key communication skills: *Engaging* patients and their caregivers to participate in consultations and decisions regarding the patient’s care, *Responding* to patients’ concerns, *Informing* patients about treatment choices, and *Framing* prognosis using balanced information about best and worst case scenarios. In other settings, these skills have been associated with improvements in psychological well-being, quality of life, symptoms, adherence to treatment, patient satisfaction, and caregiver bereavement, as well as reductions in racial and ethnic disparities [1,12,22-24]. If our hypotheses are supported, patients and their caregivers will be better informed, less psychologically distressed, and better able to participate in discussions about prognosis and treatment choices. As a result, they will be more likely to make decisions that lead to improved quality of life, a greater sense of peace, and better quality of death.

### Ecological framework

VOICE was designed to test an innovative synergistic intervention for improving communication between patients with advanced cancer and oncologists. Nearly all communication intervention studies have been individually focused on either patient or clinician behavior, or have used third parties to broker communication [25]. Such efforts have been insufficient to improve shared understanding and patient well-being. Interventions designed to help patients with serious illnesses to ask questions and express concerns have been more effective when physicians also encourage active patient participation [22,26,27]. Brief physician training can improve some aspects of communication, such as empathy. More intensive interventions have been necessary to reach broader communication goals [12,27,28]. Mindful that it may be challenging for physicians to set aside 2–3 days from a busy practice to complete training workshops, the VOICE intervention provides brief, highly-skilled and individually-tailored training embedded within the oncology practice. Further, previous research and pilot work undertaken for this trial have noted physician frustration and patient dissatisfaction when assertive patients encounter physicians who are not adequately prepared [12,22]. This suggests the need to intervene simultaneously with patients and physicians. Our trial, based on ecological theory, is the first rigorous test of an intervention that intercedes at the level of the physician-patient-caregiver relationship.

**Figure 1:**
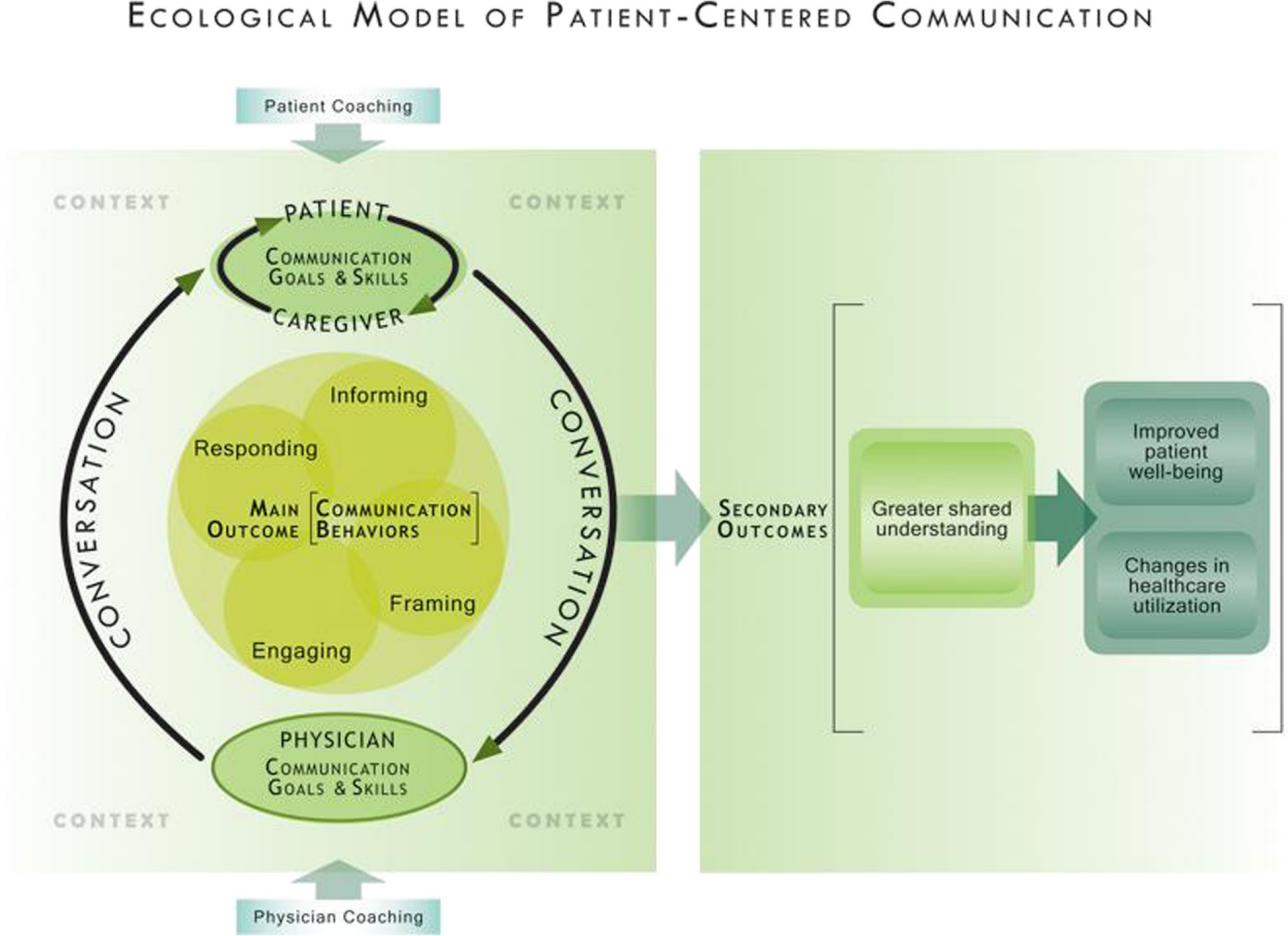
Ecological model of patient-centered communication.

Street’s ecological theory of patient-centered communication (see Figure 1) [29] was used to derive the aims, intervention, and outcome measures for this trial. Ecological theory is a systems-oriented theory [30]. As such, it suggests that clinical communication research should address multiple levels, namely the mutual interactions between physicians and patients as well as the social and clinical contexts, rather than merely targeting the individual’s communication behavior. On the interactional level, ecological theory suggests that two factors – patients’ and caregivers’ assertive behaviors, and physicians’ facilitative behaviors – interact to reinforce patients’ ongoing participation in discussions regarding their care over time [27,31]. As a result, these discussions will more closely address patients’ wishes and concerns, and patients will get more useful information, support, and empathy as well as participate in decision making to a greater degree [32,33].

Ecological theory has successfully predicted that (a) assertive patient behaviors such as making requests, asking questions, and expressing opinions generally promote physicians’ patient-centered behaviors [29,32,34], (b) physicians’ facilitative behaviors such as addressing patients’ concerns and helping patients to participate in care reinforce patient assertiveness [13,35], and (c) when caregivers are present in consultations, they can align the efforts of clinicians and patients towards common communication goals and help patients to understand and use relevant information [36,37]. Ecological theory also partially explains paradoxical results in which some outcomes, such as patient satisfaction and physician frustration, worsen when patients are trained to communicate assertively and physicians are unprepared for change [22,34]. Because of these findings, our outcome measures consider both individual communication behaviors and mutual interactions.

Importantly, ecological theory has been informed by experimental research on how *Framing* affects patient understanding and decision-making [38,39]; as well as how *Informing* patients about treatment options, *Responding* to emotional concerns, and *Engaging* patients by exploring assumptions and beliefs [40-42] help patients to be more active participants in care [43,44]. Ecological theory emphasizes that patients’ desire for information should be considered by first asking patients what they wish to know and in what format, providing desired information, then checking understanding, a paradigm known as the “Ask-Tell-Ask” approach [45]. Bidirectional exchange of tailored and desired information thus leads to better shared understanding, prepares patients to be involved in decisions, and promotes trust that important information is not being withheld [17,26].

### Current investigation

Drawing upon this ecological framework, the VOICE intervention is designed to help physicians, patients with advanced cancer, and caregivers to communicate more effectively about issues likely to influence decision making. In doing so, this investigation brings together research on communication and medical decision making, which have historically been two separate approaches addressing common issues but without a common frame of reference. Moreover, the intervention targets patients diagnosed with incurable cancer *before* they become critically ill, anticipating the need for information and strong patient-physician relationships as the illness progresses. We hypothesize that, relative to care as usual, the intervention will improve physician-patient-caregiver communication about prognosis and treatment choices (Aim 1a), improve the physician-patient relationship and increase shared understanding of prognosis (Aim 1b), improve patient well-being (Aim 2), and affect health services utilization by both reducing the number of aggressive interventions that may undermine the quality of life in the last weeks of life and increasing the use of guideline-concordant palliative care and hospice services (Aim 3).

## Methods/design

### Study design

The study is a cluster RCT conducted in multiple oncology practices and cancer centers in the Rochester/Buffalo, NY and Sacramento, CA regions, designed to evaluate the effects of a theory-based intervention to improve communication between oncologists and patients with advanced cancer and their caregivers. The methods were developed in collaboration with Phyllis Butow, Martin Tattersall, Adam Walczak and colleagues, who are conducting a parallel study in Sydney, Australia called *Conversations with Your Doctor*: *Making the Most of Medical Consultations*. That study incorporates similar interventions but a different study design. The VOICE RCT incorporates many elements of effectiveness studies, such as broad eligibility criteria, usual-care controls, tailored interventions, patient-oriented outcome measures, and intention-to-treat analyses. The study design and all consent forms have the approval of the Institutional Review Boards at each institution.

**Figure 2:**
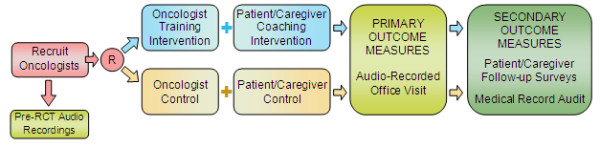
VOICE study design.

The study procedures (see Figure 2) are separated into two phases. Phase 1 involves preparation, physician recruitment, piloting, and pre-randomization data collection of physicians’ communication behaviors. Three Phase 1 patients and their caregivers are recruited for each physician. Each of these patients has one office visit audio recorded, allowing us to assess physicians’ baseline communication behaviors for potential use as a covariate in the RCT analyses. Phase 1 patients and caregivers also complete pilot versions of some study measures pre- and post- office visit to inform finalized versions of the measures for the RCT.

Phase 2 is the cluster RCT. Physicians are the unit of randomization and are randomly assigned to the intervention or control condition. Up to seven Phase 2 patients (and caregivers) are recruited per physician. Patients and their caregivers are randomly assigned by proxy: patients of physicians in the intervention condition are assigned to the intervention, and patients of physicians in the control condition are assigned to the control group. Patients complete measures at study entry, participate in the intervention or control condition, agree to have an oncology office visit audio recorded, and complete follow-up measures immediately after the office visit, approximately 2–4 days after the office visit, and every 3 months for up to 3 years or until death. Caregivers, where available, also participate in the study and complete measures periodically, including measures one-month post-mortem.

Following completion of Phase 2, data analysis will ensue. After the study has closed, physicians from the control group will be offered the opportunity to receive the intervention, both as an incentive to participate and to further their professional development. In addition to the primary VOICE trial, additional studies are using the procedures described here to examine patient, caregiver, and oncologist emotional processes [46] that drive decision making, and the effect of the intervention on caregiver bereavement outcomes. Prior research [24] suggests that improvements in end-of-life care can mitigate bereavement-related morbidity.

### Selection of study sites

In the Rochester region, oncologists (*n* = 25) were recruited from academic and private practices in western New York; recruitment is complete. In the Sacramento region, all oncologists are recruited from the UC Davis Comprehensive Cancer Center (*n* = 14), and recruitment is nearly complete. One Rochester physician withdrew from the study prior to any data collection due to lack of time. We anticipate a total of 40 oncologists.

### Eligibility, recruitment, consent, and randomization

Ethical approval was obtained from the IRBs of the five affiliated institutions where the study is being conducted. All participants (i.e., patients, caregivers, and oncologists) complete written informed consent documents. Specific inclusion and exclusion criteria are presented in Table 1, and participant eligibility is verified immediately prior to completing baseline study measures.

**Table 1:** Inclusion and exclusion criteria for oncologists, patients, and caregivers

**Participant**	**Inclusion criteria**	**Exclusion criteria**
Oncologist	● Currently in clinical practice at participating institutions	● Non-physicians and physicians who are not oncologists
	● Oncologist that cares for patients with solid tumors	● Oncologists who exclusively care for patients with hematological malignancies
	● Not planning to leave the practice during the next 6 months	
Patient	● Currently a patient of an enrolled physician	● Anticipating bone marrow transplantation or diagnosed with leukemia or lymphoma
	● Age 21 years or older	
	● Diagnosis of Stage III or IV solid (non-hematological) cancer ^a^	● Unable to complete orally-administered surveys in English
	● Able to understand spoken English (study personnel to read materials to low literacy patients)	● Hospitalized or in hospice care at recruitment or for baseline measures
Caregiver	● Caregiver of a patient currently enrolled in the study	● Unable to complete orally-administered surveys in English
	● Age 21 years or older	● Supported the patient primarily through a professional role (e.g., clergy)
	● Able to understand spoken English (study personnel to read materials to low literacy caregivers)	

^a^ Patients with Stage III cancer are included only if they have a limited prognosis. Their oncologist must affirm that they “would not be surprised” if the patient died within 12 months, thereby excluding patients with potentially curable Stage III cancers, such as Stage IIIc testicular cancer or Stage IIIa colon cancer.

#### Oncologists

Medical oncologists who care for patients with solid (non-hematologic) cancers are solicited for participation through presentations about the study at grand rounds and faculty meetings, or through personal contacts via cancer center directors or project investigators. Interested oncologists then meet with study personnel (e.g., project manager, research assistant, or research oncologist) to achieve a clear understanding of all study components, provide written consent, and complete the baseline surveys. The consent document seeks each oncologist’s agreement to (a) guide recruitment efforts among their patients by determining eligibility, (b) participate in an educational program to enhance their communication skills, (c) audiotape one clinic encounter per enrolled patient, and (d) complete brief surveys at baseline, after each audio-recorded office visit, and the study conclusion. Oncologists are told “the purpose of this study is to assess the impact of brief educational interventions on clinical care and outcomes of patients with cancer and their caregivers. Outcomes of interest include communication between patients, caregivers, and physicians, as well as patient well-being and health services utilization.” Oncologists receive $600 for completing surveys and identifying eligible patients for the study.

#### Patients

Research assistants identify all patients by working closely with participating oncologists and their clinic staff to review clinic rosters in detail to ascertain that all potentially eligible patients are identified. Depending on the site and differing IRB requirements, potentially eligible patients are either (a) approached by physicians or practice nurses and then by the research assistant or (b) sent a letter, a study brochure, and an opt-out card. Patients approached by physicians or clinic staff are asked if they would be willing to speak to a research assistant to learn more about the study. Patients who receive recruitment materials by mail are contacted via phone by a research assistant if an opt-out card is not returned to the research office within 7 days. The research assistant provides them with study information, and gives them time to deliberate about participation and ask questions. The screening and consent process continues until the research assistant feels that the potential participant fully understands all aspects of study involvement. The research assistant then schedules a time to meet and obtains written consent from those who voluntarily wish to enroll, using IRB-approved consent forms. Patients are reminded that they can opt out at any time. All patients are asked to complete baseline surveys and to give permission to have one of their oncology office visits audio recorded. The research assistant orally administers the baseline surveys as well as other study materials as needed. If consent documentation and baseline survey measures are completed in separate visits, eligibility criteria are reviewed immediately prior to survey administration to ensure continued eligibility.

Phase 1 patients complete two sets of orally-administered surveys, one before and one after the audio-recorded office visit with their oncologist. Phase 2 patients (a) have one audio-recorded office visit with their oncologist, (b) complete surveys prior to and immediately after their office visit, then 2–4 days after the office visit, and every three months for up to three years, and (c) give permission for research staff to access their medical records to ascertain their use of health services such as emergency department visits, hospital admissions, cancer treatments, and community-based nursing services. The initial survey takes up to 60 minutes with later surveys taking anywhere from 10 to 30 minutes. Patients receive $15 for each set of surveys, for a maximum of $30 in Phase 1 and $180 in Phase 2.

#### Caregivers

All patients are asked to identify a “family member, partner, friend or someone else who is involved with your health care issues, for example, someone who you talk to about personal issues including medical decisions or who comes to doctor appointments with you. This person may also help with routine day-to-day activities, like transportation or paperwork.” The term “caregiver” is used to describe these persons for scientific purposes, and patients are not required to self-identify individuals as such. Patients are asked to identify up to three potential caregivers, rank them in order of the likelihood that they will attend oncology office visits, and provide permission to contact the primary caregiver (or others caregivers, if needed) and provide them with a study brochure.

Once identified, the research assistant independently approaches the caregiver in person or by telephone and asks if they would be willing to participate and provide written consent. Caregivers often accompany patients at the time of the patient consent. The research assistant reviews the consent form in detail with the caregiver and advises them that they can opt out of the study at any time. Caregivers are told the same study details as the patients and complete surveys at approximately the same time intervals as the patients. Caregivers are asked for permission to be contacted in the event that the patient dies within three years of enrollment in order to meet with a research assistant (or speak on the telephone if preferred) to complete a survey. The survey is completed approximately one month after the death of the patient. The research assistant responds to caregivers’ questions until it is clear that the caregiver has full comprehension of the consent form and their involvement in the study. Caregivers receive $15 for each set of surveys, for a maximum of $30 in Phase 1 and $180 in Phase 2.

#### Randomization

A stratified block-randomization scheme is used to assure balanced assignment by clinic site and cancer focus. Oncologists are grouped into a site according to their health center, clinic, or practice of employment. Within each site, oncologists are randomly assigned approximately evenly across the treatment and control conditions. Sites with a single oncologist are grouped with a similar site for randomization purposes. Oncologists are also categorized by their cancer focus, including breast-cancer oncology (≥50% of patients have diagnoses of breast cancer) and non-breast cancer oncology groups. Within these areas of focus, oncologists are randomly assigned approximately evenly across the treatment and control conditions. This accounts for any biases that may be introduced by the low prevalence of breast cancer in men and the potential that breast cancer patients may be more “activated” than patients with other cancers. For each site/focus combination in the study, separate sequences of random numbers have been generated for use in assigning oncologists to the intervention and control groups. To preserve blinding, assignment to the treatment or control conditions is maintained by the study statistician and not explicitly revealed to transcriptionists or coders of the audio-recorded office visits.

### Visit procedures

For the initial baseline surveys, a time is arranged to meet with the patient and caregiver, either together or separately, in a private area based on the participant’s comfort and preference. This may include a meeting room in the cancer center, the infusion suite, the participant’s home, or a local coffee shop with private areas. Once both the patient and caregiver have completed these surveys, the research assistant answers any questions and reviews the next steps of the study, the audio-recorded office visit and post-visit survey. For that visit, the research assistant usually meets the patient and caregiver in the physician’s waiting room and accompanies them to the clinic room, turns on and places two audio recorders in the room, and leaves. If another person attends the visit, the research assistant obtains verbal permission to record them. The research assistant collects the recorders after the office visit and meets with the patient and caregiver to administer brief surveys. This completes Phase 1. In Phase 2, the scenario is identical, except that the patients and caregivers in the intervention arm receive a 1-hour coaching session prior to the office visit. After the audio-recorded office visit, the patient completes a survey, and the research assistant explains to the patient and caregiver that they will be contacted in 2–4 days by phone to answer some additional questions. The patient and caregiver are also called three months after the audio-recorded visit to complete surveys and every three months thereafter for up to three years.

### Description of the intervention and control conditions

#### Intervention condition

Oncologists randomized to the intervention arm participate in a multifaceted tailored educational intervention involving standardized patients instructors (SPIs). Patients and caregivers complete a coaching intervention to facilitate prioritizing and discussing questions and concerns.

**Oncologist training** Oncologists completing the intervention meet with SPIs for two in-office educational outreach sessions [47], including a 60-minute training session and a 45-minute booster session [48]. At the first session, the SPIs show them a 15-minute DVD created by the study investigators specifically for this project, which presents actual clinical examples to outline key skills in discussing prognosis and treatment choices with patients with advanced cancer and their caregivers. Oncologists receive a copy of the DVD to keep and receive a 1-page summary of evidence-based guidelines for communication in advanced cancer [13]. They are also given a *Communication Guide* “reminder” card prompting them to discuss topics, such as prognosis and symptoms that appear on the patients’ Question Prompt List, such as prognosis and symptoms (see below, QPL).

After viewing the DVD, the oncologist is asked to select one or two of the key skills to practice with two SPIs who portray a 60-year-old man with incurable cancer and his wife. A few days prior to the session, the oncologist is sent a medical “chart” to review in anticipation of the SPI visit, containing a comprehensive prior consultation note which includes radiographic and laboratory results. In the “chart,” the patient is described as having metastatic colon cancer with progression despite one course of state-of-the art chemotherapy, and intolerance of a second course of chemotherapy. Current symptoms include severe diarrhea, nausea, and fatigue. The SPIs present as facing important treatment decisions over the next few weeks, while having an unclear idea of prognosis. The SPIs engage the oncologist in a role play exercise with specific tailored feedback on the key communication skills described in the DVD; oncologists are then given the opportunity to rehearse areas of difficulty. Oncologists also receive a follow up letter from the SPIs that includes a version of the communication guide with individually-tailored comments. The guide shows the four key skills discussed in training and summarizes what was agreed to by the oncologist and SPIs during the session, such as the physician’s demonstrated areas of strength, as well as those areas jointly identified as challenges for further growth. One month later, oncologists complete a 45-minute reinforcement session, which uses a similar format. Specifically, the same SPIs return for a simulated follow-up visit, where the cancer has “progressed” despite third-line treatment. Key communication skills highlighted in the DVD and SPI feedback (see Table 2) were chosen based on ecological theory and evidence that they (a) promote discussions of prognosis and treatment choice, (b) can be taught in brief interventions, and (c) are associated with patient trust and lower anxiety [13,49-51].

**Table 2:** Oncologist communication behaviors targeted in the tailored educational intervention

**Behavior**	**Description of intervention**
Engaging	Oncologists are coached to (a) clarify the patient’s concerns early in the visit [52] – this corrects the tendency to address the first concern mentioned at the expense of more important issues [53], (b) acknowledge the Question Prompt List (QPL) to increase its effectiveness [26], (c) encourage questions, and (d) encourage participation in healthcare decision making [26,54,55].
Responding	Emotional expression and empathy are uncommon in oncology consultations [51,56,57]; therefore, oncologists are coached to respond to the emotional components of patients’ concerns with empathy and support.
Informing	Based on recent studies [58,59], oncologists are coached to use an “Ask-Tell-Ask” protocol – asking patients about their wishes regarding information about prognosis and treatment choices, providing desired information in a desired format, and then checking patient understanding.
Framing	Based on recent studies [6,58], oncologists are trained to present information for both optimistic or “best case” and pessimistic or “worst case” scenarios. Balanced information appears to better align patients’ and physicians’ efforts by reducing bias introduced by one-sided presentation of data.

**Patient and caregiver coaching** Patients and caregivers completing the intervention meet with a coach trained to facilitate health communication. During the coaching session, the coach gives each patient and caregiver a Question Prompt List (QPL), which is organized in a booklet called *My Cancer Care*. The QPL includes questions about diagnosis, prognosis, treatment options, symptom management, transitions in care, self-care, and family needs. Sample questions include, “What are the pros and cons of further treatment for my cancer?” and “How can I help my family and children understand what is happening?” The QPL was developed in Australia, adapted for the United States, and piloted simultaneous in both countries [60]. Using the QPL as a guide, patients are coached to (a) identify and prioritize 2–3 personally relevant questions on the list, (b) ask these questions during the visit, (c) ask their oncologist for clarification when they do not understand, (d) express desire to participate more actively in discussions about prognosis and treatment choices, and (e) prepare for the future [22,26,54,61]. These skills promote the same goals as the physician intervention. The coach makes follow-up phone calls at monthly intervals for up to three months to reinforce the coaching intervention and address patients’ concerns [62]. The coaches include a nurse and social workers with healthcare backgrounds, similar to prior studies [61]; they do not provide disease-specific information. All Rochester and UC-Davis coaches participate in local training, video conferences, and a 3-day intensive training, using methods and materials similar to our previous studies [63]. All intervention sessions are audio-recorded and progress notes are written after each of the 3 coaching follow-up telephone calls. The coaches hold weekly conference calls to fine-tune, discuss, and review their coaching sessions.

#### Control condition

The control group involves care as usual. Oncologists as well as patients/caregivers meet with the research assistants to complete the same surveys as intervention participants but receive no training.

### Measures

#### Oncologist-patient-caregiver communication

The audio-recorded office visits for all participants are coded for each of the four domains of communication behaviors hypothesized to be affected by the intervention: *Engaging*, *Responding*, *Informing*, and *Framing* (see Table 2). The Active Patient Participation Coding (APPC) [32] scheme is used to measure *Engaging* communication behaviors, such as patient assertive behaviors and oncologist facilitative behaviors, that promote patient participation in the decision-making process. The Verona VR-CoDES system [64] is used to code for sequences of *Responding* to emotion, such as patient expression of emotional cues and concerns and oncologist responses to these expressions of emotion. The Prognostic and Treatment Choices (PTCC) [65] system is used to code *Informing* behaviors, such as the oncologist addressing patients’ wishes for information regarding prognosis and treatment choices. The Optimism/Pessimism subscale of the Framing of Prognostic Information (FPI) [6] system is used to code *Framing* behaviors, such as oncologists’ balance in expressions of optimistic and pessimistic perspectives about “what to expect.” Several exploratory measures assess the level of patient and caregiver question asking [26,54] and the degree of shared decision making [66,67].

#### Patient survey measures

The survey measures completed by patients in Phase 2 are outlined in Table 3. Several scales measure aspects of the quality of interactions between physicians and patients. The quality of the *Physician*-*Patient Relationship* is measured with The Human Connection (THC) [68] survey. Characteristics of the *Physician*-*Patient Interaction* are measured with the Health Care Communication Questionnaire (HCCQ) [69], the Mishel Uncertainty in Illness Scale (MUIS) [70], and the Information Preference Scale (IPS) [71]. *Patient Communicational Self*-*Efficacy* is measured using an adaptation of the Perceived Efficacy in Patient-Physician Interactions (PEPPI) [43] survey. The *Physician*-*Patient Conversations* survey assesses topics discussed in recent oncology office visits, such as prognosis, end-of-life care, and emotional issues, supplementing information obtained from the single audio-recorded oncology office visit.

**Table 3:** Survey measures completed by patients in the RCT

**Domain**	**Measures**	**Study entry**	**Post- visit**	**2-4 day follow-up**	**Quarterly follow-up**
Demographics	Gender, age, race/ethnicity, SES, relationship status, religion	X			
Physician-Patient Relationship	THC	X		X	X^a^
Physician-Patient Interaction	HCCQ + MUIS + IPS		X		
Patient Communicational Self-Efficacy	PEPPI	X		X	X^a^
Physician-Patient Conversations	Topics discussed in recent medical encounters	X			X
Preferred Decision Role	CPS	X		X	
Actual Decision Role	Modified CPS		X		X
Treatment Preferences	Preferences for experimental treatments, life support, palliative care	X		X	
Illness Acceptance	PEACE	X			X
Well-being	MQOL + FACT-G	X			X
Prognostic Forecasting	Estimate of prognosis	X		X	

*Note*. *SES*, Socioeconomic status; *THC*, The human connection scale; *HCCQ*, Health care communication questionnaire; *MUIS*, Mishel uncertainty in illness scale; *IPS*, Illness preference scale; *PEPPI*, Perceived efficacy in patient-physician interaction; *CPS*, Control preferences scale; *PEACE*, Peace, equanimity, and acceptance in the cancer experience; *MQOL*, McGill quality of life; *FACT*-*G*, Functional assessment of cancer therapy – general.

^a^ Administered at only the first quarterly follow-up.

Several scales relate to patient attitudes, values and beliefs. Patients’ *Preferred Decision Role* is measured with the Control Preferences Scale [72], which is compared to an adapted version assessing patients’ *Actual Decision Role* in oncology office visits. The *Treatment Preferences* survey measures preferences for experimental treatments, life support, and palliative care, in the event that no further anti-cancer treatments would be helpful. *Illness Acceptance* is measured with the Peaceful Acceptance subscale of the PEACE [7]. *Prognostic Forecasting* is measured using items [5] assessing patients’ beliefs about their chances of living two years and chances of being cured.

Finally, *Well*-*being* is measured with the Global Quality of Life, Psychological Well-being, and Existential Well-being subscales of the McGill Quality of Life (MQOL) [73] survey as well as the Physical Well-being and Social/Family Well-being subscales of the Functional Assessment of Cancer Therapy – General (FACT-G) [74] survey.

Each of these surveys has been tailored to improve clarity and relevance, reduce respondent burden, and reflect American English. Most of these measures have been piloted with patients in Phase 1 and refined as needed to reduce ceiling and floor effects or further improve item wording.

#### Caregiver survey measures

The survey measures completed by caregivers in Phase 2 are outlined in Table 4. Measures of the *Physician*-*Caregiver Relationship*, *Physician*-*Caregiver Interaction*, and *Caregiver Communicational Self*-*Efficacy* have been adapted from patient versions of the same scales. Using measures adapted from patient versions, caregivers also provide informant ratings of *Patient Treatment Preferences*, *Patient Illness Acceptance*, and *Patient Well*-*being*, and complete a measure of *Prognostic Forecasting*. They report on *Patient Quality of Death* via qualitative questions about the healthcare decision-making process, the Quality of Life Near Death (QOLND) [75] survey, and items from the Quality of Death Long-Term Care – Cognitively intact (QOD-LTC-C) [76] survey. As with the patient measures, each of these surveys has been tailored for the current study.

**Table 4:** Survey measures completed by caregivers in the RCT

**Domain**	**Measures**	**Study entry**	**2-4 Day follow-up**	**Quarterly follow-up**	**Post- mortem**
Demographics	Gender, age, race/ethnicity, SES, relationship with patient	X			
Physician-Caregiver Relationship	THC		X		X^b^
Physician-Caregiver Interaction	HCCQ		X		
	MUIS		X	X^a^	
Caregiver Communicational Self-Efficacy	PECPI	X		X^a^	
Patient Treatment Preferences	Caregiver’s beliefs about patient preferences for experimental treatments, life support, palliative care	X		X^a^	
Patient Illness Acceptance	PEACE	X			X^b^
Patient Well-being	MQOL + FACT-G	X			X^b^
Prognostic Forecasting	Estimate of patient’s prognosis	X	X		
Patient Quality of Death	Qualitative questions + QOLND + QOD-LTC-C				X^b^

*Note*. *PECPI*, Perceived efficacy in caregiver-physician interaction; *QOLND*, Quality of life near death; *QOD-LTC-C*, Quality of death long-term care – cognitively intact; other acronyms defined previously (see Table 3).

^a^ Administered at only the first quarterly follow-up.

^b^ Retrospective rating of the death/dying experience, rather than the current moment.

#### Oncologist survey measures

The survey measures completed by physicians are outlined in Table 5. *Communication Skills* are measured with a pilot-tested survey derived for this study, which assesses perceived skills in discussing prognosis and end-of-life issues, such as giving bad news, expressing empathy, and discussing referrals to palliative care. *Decision Making Skills* are measured with a survey adapted from a prior measure [72] in order to assess physician comfort with decision making across varying levels of patient involvement (e.g., physician makes the decision, patient makes the decision, shared decision making). Several measures have been adapted from the patient self-report surveys: physician beliefs about *Patient Treatment Preferences*, physician beliefs about *Patient Illness Acceptance*, and *Prognostic Forecasting* about patients’ prognoses (e.g., chances of a cure, chances of living two years, patients’ understanding thereof). *Patient Disease Status* is assessed via several survey items, which supplement information from patient medical records.

**Table 5:** Survey measures completed by physicians

**Domain**	**Measures**	**Study entry**	**Post-visit**	**Study conclusion**
Demographics	Age, race/ethnicity, gender, training background, practice characteristics	X		
Communication Skills	Skills in discussing prognosis and end-of-life care	X		X
Decision Making Skills	Comfort with decision making across varying levels of patient involvement	X		X
Patient Disease Status	Cancer site, progression, treatment planning		X	
Patient Treatment Preferences	Physician’s beliefs about patient preferences for experimental treatments, life support, palliative care		X	
Patient Illness Acceptance	PEACE		X	
Prognostic Forecasting	Physician’s estimate of prognosis, and beliefs about patient’s estimate of prognosis		X	

*Note*. *PEACE*, Peace, equanimity, and acceptance in the cancer experience.

#### Medical chart abstraction

The death of participating patients is ascertained by checking in regularly with participating oncologists, through scheduled follow-up sessions with patients and caregivers, by reviewing death notices (obituaries) in the local press, and by periodically reviewing electronic health records. Once a death is identified, study staff identify all emergency department visits or hospital overnight stays occurring in the last 30 days before death and abstract the corresponding medical records for the following data elements: (1) date of death, (2) dates and circumstances of attendance at a hospital, emergency department, and intensive care unit, (3) use of intubation, cardiopulmonary resuscitation (CPR), hemodialysis, permanent enternal feeding tube, and chemotherapy, (4) involvement of hospice and palliative care services, and (5) completion of Do Not Resuscitate/Do Not Intubate (DNR/DNI) forms and Advanced Directives. Outpatient records are abstracted to identify any chemotherapy agent given in the last 14 days before death and any new chemotherapeutic regimen started in the last 30 days before death, the absence of which indicate higher quality end-of-life care [77].

### Implementation fidelity

Both the oncologist and patient/caregiver components of the intervention are monitored for fidelity. For the oncologist training sessions, all audio recordings are reviewed by all SPI trainers. For the patient/caregiver component, audio recordings are reviewed for each coach’s first five coaching sessions, every subsequent session until >95% fidelity, and at least every fifth session thereafter, including in-person and phone sessions. To assess fidelity and ensure standardization of survey administration procedures, each research assistant’s first five sessions are monitored by direct observation and reinforced every 4–6 months during data collection. In addition, audio-recordings of qualitative survey items are reviewed thereafter as needed.

### Sample size determination

This is a stratified cluster randomized study, with the physician as the unit of randomization. Based on prior studies, we have made the following assumptions: physician attrition 0-3%, patient attrition <5% for audio-recordings, and 10%, 30% and 35% for the 2–4 day, 3-month, and 6-month post-visit patient surveys [78,79], 80% patient mortality during 3-year follow up, availability of 85% of caregivers for post-death interviews [3,24], availability of 90% of medical records for audit [3,5,80], no differential attrition between the intervention and control groups, and an intraclass correlation coefficient (ICC) of .10 or less for within-physician clustering on patient and caregiver survey measures [81,82].

Prior work found that activation training increases physician communication behaviors two to three fold [26,28,29]. This equates to an effect size of 2.0 standard deviation (*SD*) units. Thus, for our primary outcome (Aim 1a), we expect a 2.0 *SD* improvement across each communication measure; 0.5 *SD* is clinically significant. Power is based on a single measurement of communication during the oncology office visit. With at least 19 oncologists in each group, 7 patients per oncologist, and an ICC of .10, the minimum detectable effect size is 0.50 *SD*.

For our survey outcomes (Aims 1b & 2), we rely on observational data [83] indicating effect sizes of 0.40 to 0.70 *SD* for the relationship between communication and patient well-being. With an intervention we would expect these differences to be larger. The power analysis takes advantage of a repeated-measures design [84]. For patient data, we assume one pre-intervention measure and two post-intervention measures, and an average correlation among repeated measures of .50. Our proposed sample size is sufficient to detect an effect size of 0.40 *SD* with power of .80. For our utilization outcomes, we rely on data that suggest 3- to 8-fold differences in use of aggressive treatments during the final week of life between patients who have had discussions compared to those who have not [3,5]. Thus, the study will be adequately powered, even considering attrition.

### Planned analytic approach

This is a cluster-randomized trial, where our primary communication outcomes (Aim 1a) are measured at the level of the physician-patient dyad and our secondary outcomes (Aims 1b, 2, & 3) are measured at the level of the patient. Analyses are based on published guidelines for group (cluster) RCTs [85], in this case, clustering at the site and physician level. Prior to hypothesis testing, preliminary analyses will examine whether random assignment produced comparable groups in terms of patient disease status, patient well-being, and pre-RCT (Phase 1) physician communication styles. Any confounders will be included in remaining analyses. Hypothesis testing will involve comparisons of the two randomized groups. Since the patients are nested within oncologists, methods for panel data will be applied, and random effects for physicians may be added to account for the within-physician correlations of each dyad. We will use both generalized estimating equations (GEE) and generalized linear mixed-effects models (GLMM). The primary outcomes (Aim 1a) are unlikely to require substantial attention to attrition, since measurement occurs shortly after randomization, and will not require methods for repeated measures. Hierarchical linear models with nested random coefficients will be applied where secondary outcomes that are measured repeatedly (Aims 1b, 2, & 3). As missing values are likely, we will examine the nature of the missing data and use weighted GEE (WGEE), or multiple imputation and sensitivity analyses, if necessary. In assessing significance across multiple tests, we will use procedures to control the false discovery rate (FDR) at 5% within sets of related analyses [86].

## Discussion

The VOICE Study is a multi-site randomized controlled trial designed to facilitate communication about prognosis and healthcare decision making among oncologists, patients with advanced cancer, and their caregivers (defined as family, friends, or other non-professionals who are involved in their care). The investigation is unique in bringing together research on patient-centered communication and medical decision making, intervening with both physicians and patients/caregivers, and its upstream focus, targeting patients with advanced cancer before they are critically ill.

The proposed intervention anticipates future needs. In the future, patients with advanced cancer will have more complex and numerous treatment options, including biologicals, genetically-tailored therapies, and new devices. This complexity will present challenges for physicians in providing the best possible quality of care for incurable conditions while maintaining realistic hope. Thus, the need for effective communication in the context of incurable cancer will increase. Furthermore, effective communication is integral to current health reform efforts. Programs to enhance communication will potentially be met favorably by health systems, insurers, and federal agencies who value the patient-physician relationship and by the public who value having control over their own care. The proposed intervention is also scalable for dissemination using new technologies. Currently, a trained Standardized Patient Instructor (SPI) and coach can visit dozens of practices at a reasonable cost, and training materials and question prompt lists can be adapted for the Web and embedded in electronic health records. In the future, increasing bandwidth will permit adaptation of the SPI and coaching interventions to live web-SP technology [87,88], offering live training to clinicians and patients regardless of location.

There have been several meaningful challenges in implementing VOICE. In order to recruit physicians, it has been important to contact senior physicians within each practice to garner support for the study and work closely with nursing and office staff in order to minimize the burden of physician participation. To maintain the fidelity of the intervention, well-trained supervisors of SPIs and coaches are needed, who can provide timely and meaningful feedback to those implementing the intervention based on a detailed review of audio recordings of intervention sessions. Measurement issues also required attention, as several scales needed to be adapted (a) from British or Australian English to American English, (b) to improve the consistency of response scales to reduce cognitive load, (c) to increase the number of response options to mitigate ceiling and floor effects in our sample, and (d) to reduce the number of items to prevent respondent burden in a sample of seriously ill participants. Inclusion and exclusion criteria also warranted attention; for example, a patient with two malignancies, one hematologic and one solid, was excluded because the hematologic malignancy was active and progressing.

The pragmatic design of the intervention should facilitate dissemination of the program if proven effective. The main challenges of the study have been related to implementation issues (e.g., SPI and coach training and monitoring) and study design issues (e.g., respondent burden, IRB issues, and opt-out cards). The former presented few difficulties, and the study design challenges would not be present if the program were to be widely disseminated.

## Competing interests

The authors declare that they have no competing interests.

## Authors’ contributions

RME and RLK are dual-PIs of VOICE and developed the original study protocol. MH, RME, KF, PRD, RG, SGM, SP, DT, AV, CC, GE, CF, DG, BH, BL, LL, MR, PS, and RLK planned, coordinated, and conducted the study. PCW and WT provided statistical and methodological support, and PCW oversaw data management. PNB, PRK, AW, and ALB provided consultation aimed at improving the study design. MH coordinated the survey measurement group and drafted the initial version of the manuscript. All authors reviewed and approved the final version of manuscript.

## Pre-publication history

The pre-publication history for this paper can be accessed here:

http://www.biomedcentral.com/1471-2407/13/188/prepub
